# Native Biocrust Cyanobacteria Strains Showing Antagonism against Three Soilborne Pathogenic Fungi

**DOI:** 10.3390/pathogens13070579

**Published:** 2024-07-11

**Authors:** Pilar Águila-Carricondo, Raúl Román, José Ignacio Marín-Guirao, Yolanda Cantón, Miguel de Cara

**Affiliations:** 1 Department of Chemical, Energy and Mechanical Technology, Universidad Rey Juan Carlos, C/Tulipán s/n, 28933 Móstoles, Spain; 2 School of Life Sciences, University of Nevada-Las Vegas, Las Vegas, NV 89154-4004, USA; jrf979@gmail.com; 3 IFAPA-La Mojonera, Camino San Nicolás n.1, 04745 La Mojonera, Spain; josei.marin@juntadeandalucia.es; 4 Centro de Investigación de Colecciones Científicas de la Universidad de Almería (CECOUAL), University of Almería, 04120 Almería, Spain; ycanton@ual.es

**Keywords:** biocontrol, cyanobacteria, growth phase, *Nostoc*, *Phytophthora*

## Abstract

The biocontrol potential of three native soil cyanobacteria from biological soil crusts (*Nostoc commune*, *Scytonema hyalinum*, and *Tolypothrix distorta*) was tested by means of in vitro mycelial growth inhibition assays for eighteen cyanobacteria-based products against three phytopathogenic soilborne fungi (*Phytophthora capsici*, *Pythium aphanidermatum*, and *Fusarium oxysporum* f. sp. *radicis-cucumerinum*). Three cyanobacteria-based production factors were considered: (i) cyanobacterium strain, (ii) cyanobacterial culture growth phase, and (iii) different post-harvest treatments: raw cultures, cyanobacterial filtrates, and cyanobacterial extracts. Results showed that any of the factors considered are key points for successfully inhibiting fungal growth. *N. commune* showed the highest growth inhibition rates for the three phytopathogens; stationary phase treatments produced higher inhibition percentages than logarithmic ones; and all the post-harvest treatments of *N. commune* at the stationary phase inhibited the growth of *P. capsici*, up to 77.7%. Thus, *N. commune* products were tested in planta against *P. capsici*, but none of the products showed efficacy in delaying the onset nor reducing the damage due to *P. capsici*, demonstrating the complexity of the in planta assay’s success and encouraging further research to design an appropriate scaling up methodology.

## 1. Introduction

In southeastern Spain, soils, which are usually poor in nutrients and degraded, share space with the highest concentration of Mediterranean greenhouses. In this region, the growing intensity ratio (crop growing area in relation to the greenhouse area) is around 1.45, and thus crops cover more than 50,000 ha/year [1,2]. These crops mainly include Solanaceae and Cucurbitaceae, but also Fabaceae [1,3,4]. They are a valuable source of income for the region. In 2022, Spain was one of the main European producers of pepper (49%), cucumber (29%), and tomatoes (24%) [5]. Unfortunately, this production is constantly under threat from pests, which have detrimental effects. Several soilborne diseases of horticultural crops have been reported in the area, including oomycetes such as *Phytophthora capsici* and *Pythium aphanidermatum*, as well as fungi like *Fusarium oxysporum* f. sp. *radicis-cucumerinum* [6,7,8], which are harmful root and crown rot pathogens that can survive in soils for periods of years and cause important economic losses to the agriculture sector [9,10,11].

Different strategies are adopted to control phytopathogenic fungi, which are traditionally focused on the use of chemical-based fungicides. They are normally noxious for human health and the environment, and they also kill soil microorganisms that are beneficial for plants. This, along with the EU restrictions [12] on synthetic pesticides, makes biocontrol by means of natural products extracted from plants or microbial inoculants such as cyanobacteria a promising sustainable tool [13]. Many different biofungicides have been studied for eradicating soilborne pathogens, including some cyanobacteria strains that have been proven to release a series of toxic compounds that affect fungi [14,15,16,17,18].

Cyanobacteria have been extensively studied over the last few decades in fields such as agriculture as plant growth promoters, degrading harmful agrochemicals like lindane, or in crop protection as biofungicide agents to control plant diseases caused by phytopathogens [19,20]. In vitro assays are the preliminary step to screen the effects of cyanobacteria on fungi, and hence, there exists in the literature a considerable number of studies under laboratory conditions involving mostly aquatic cyanobacteria. As successful antifungal effects strongly depend on the microalgae strain, in vitro biocontrol assays rely on the screening of different cyanobacteria. For instance, when soil cyanobacteria isolated from rice paddy fields were screened, [21], out of the 142 strains evaluated, only 9 exhibited antifungal activity. Although cyanobacteria strains from orders other than Nostocales, such as *Oscillatoria angustissima* [22], have been investigated, Nostocales have emerged as the primary focus for controlling plant diseases caused by microbes. Most of these studies were conducted using freshwater water strains [13,23], but there are also some studies evaluating the potential of soil strains [15,24,25,26]; however, the potential antifungal effect of biocrust-forming cyanobacteria is yet not well known. Biocrusts or biological soil crusts are communities of bacteria (cyanobacteria, chemoautotrophic, and heterotrophic bacteria), archaea, algae, fungi, lichens, bryophytes, and microarthropods inhabiting on or within the top few centimeters of the soil surface [27]. The interest in the latter stems from their pioneering nature, being recognized as the first colonizers of degraded drylands soils [28], and their ability to survive and colonize extreme environments. Their role in soil stabilization, nutrient enrichment, and increasing moisture has been widely demonstrated, and hence their use as soil conditioners to improve restoration outcomes is increasing [29,30]. Biocrust cyanobacteria may have the advantage that a unique application should be enough to reduce or inhibit the effects of phytopathogenic fungi because once inoculated on the soil, they colonize it and grow, becoming a natural and potentially inexhaustible source of non-toxic and environmentally friendly crop protective agents. In addition, biocrust cyanobacteria can be easily collected from the natural soil habitat, isolated, and cultured in a photobioreactor. 

Although selecting cyanobacterial strains to combat a particular phytopathogen is a critical variable to consider, other factors influencing the effectiveness of the antifungal effect include the growth phase of cyanobacteria. Results found in the literature remark that even though antifungal analytes are typically metabolized during the stationary phase or found in low quantities in the initial growth phase [31,32,33], there are exceptions: *Nostoc insulare* produces 4,4′-dihydroxybiphenyl and 9H-pyrido(3,4-b)indole toxic metabolites during its stationary phase, whereas N,N′-(4,5-dimethyl-1,2-phenylene)-bis-acetamide is detected in the linear phase [34]. *Scytonema* sp. TISTR 8208 produces an inhibitor pigment during its final linear phase, although this decreases during the stationary phase [35]. Thus, the cyanobacterium strain and its growth phase selection are interesting variables to consider when designing antifungal assays.

Since the results from in vitro studies are not conclusive because they are carried out under controlled conditions, to unequivocally establish the antifungal effect of cyanobacteria strains it is essential to perform in vivo experiments. Although some studies have reported high inhibitory effects on fungal infection by means of certain substances in vitro, the production of these substances in a natural environment still needs to be tested before incorporating them into the market as antifungal products to control plant diseases. In this sense, there is considerably less research on scaling up in vitro experiments to in planta assays, particularly involving soil cyanobacteria and none specifically targeting biocrust-forming cyanobacteria. Thus, Table 1 shows the scarcity of in vivo experiments for soil cyanobacteria inoculation applied to protect different crops against fungi, including onion, tomato, zucchini, and cucumber. Besides being essential to assess the efficacy of cyanobacteria as biocontrol agents of fungi in vivo, when scaling up the experiments from in vitro to in planta assays, the methodological approach plays a key role. In this regard, it is necessary to optimize different variables such as inoculum volume, irrigated surface, or the application in cotyledons, seeds, or roots and plant defense system activation [36] because it will determine the scaling success.

Hence, this research aims to assess the in vitro potential of three native biocrust cyanobacteria strains for controlling three soilborne phytopathogenic fungi that pose a great risk to crop production in European orchards. The study analyzed the effect of cyanobacteria strain, cyanobacteria growth phase, and three post-harvest treatments (raw culture, extract, filtrate) on the inhibitory effect. The most effective strain in the in vitro tests was additionally assessed for controlling *Phytophthora capsici* in bioassays with cucumber plants used as a model of study. 

## 2. Materials and Methods

### 2.1. Cyanobacterial Strains

Biocrust cyanobacterial strains were selected and were previously isolated from the study region [28] to maximize their specific adaptations to tolerate extreme environmental conditions [43] in the driest region in Europe. Specifically, samples of biocrust-forming cyanobacteria were collected from biocrusts covering soils with contrasting textures from two sites with different degradation levels, located in southeastern Spain (Almería): (i) “Gádor quarry”, a limestone quarry area in Sierra de Gádor (W 36°55′20″ 02°30′29″ W) consisting of a completely disturbed system with a clay loam soil texture (34% sand, 35% clay), low soil organic carbon content (about 1.12 g kg^−1^), and a total nitrogen content of approximately 0.21 g kg^−1^ [44]; and (ii) “El Cautivo”, a gully area in the Tabernas desert (N 37°00′37″ W 02°26′30″) subjected to active erosion with a silty loam (30% sand, 59% silt, and 11% clay) soil texture, an average organic carbon content in the top soil of 9.4 g kg^−1^, and a total nitrogen content of 0.97 g kg^−1^. At the two sites, the climate is semi-arid Mediterranean, with mean annual precipitation between 200 and 240 mm falling primarily in winter, and with long and dry summers [45].

Three N-fixing soil filamentous cyanobacteria strains forming part of biocrusts from the two sites were identified and isolated: *Nostoc commune* (CANT2 UAM817) and *Tolypothrix distorta* (CANT7 UAM825) from Gádor quarry (Almería, Spain), and *Scytonema hyalinum* (CAU6 UAM820) from “El Cautivo” (Almería, Spain).

### 2.2. Origin and Maintenance of Fungal and Oomycete Isolates

Pathogenic representative isolates of *Phytophthora capsici* (Mi0211), *Pythium aphanidermatum* (Mi0142), and *Fusarium oxysporum* f. sp. *radicis-cucumerinum* (FORC WI) from the IFAPA-Centro La Mojonera laboratory in Spain were used in the research. The *P. capsici* and *P. aphanidermatum* isolates were recovered from diseased sweet pepper (*Capsicum annuum*) plants cultivated in greenhouses. Oomycetes *P. capsici* and *P. aphanidermatum* are mentioned as fungi in this study. *F. oxysporum* f. sp. *radicis-cucumerinum* strain was isolated from wilted cucumbers (*Cucumis sativus*). The cultures were maintained on potato dextrose agar (PDA) (Biolife, Milan, Italy) at 25 °C in the dark and transferred every 6 months. The pathogenicity of the strains was tested prior to the research via controlled drenching inoculations on 3 true-leaves stage seedlings. Tests were performed in a growth chamber with a 14 h photoperiod (>12,000 lux), a temperature of 23–33 °C, and 40–75% relative humidity. Before planting, the cucumber seeds (cv. Super Marketer, Mascarell Semillas, Valencia, Spain) were disinfected by immersion in a sodium hypochlorite (35 g L^−1^ active chloride) solution 1:1 for 20 min; subsequently, they were rinsed with sterile water and sown on autoclaved vermiculite (121 °C, 30 min). Each pathogen was inoculated separately on six cucumber plants (pots). The inocula (propagules) concentrations were adjusted by Thomma counting cell to ca. 10^6^ UFC/plant for *F. oxysporum* f. sp. *radicis-cucumerinum* and ca. 10^4^ UFC/plant for *P. capsici* and *P. aphanidermatum*. Wilt symptoms were observed before 40 days post-inoculation in any case.

### 2.3. Cyanobacteria Culture Conditions and Post-Harvest Treatments

Each cyanobacteria strain was cultured in 250 mL Erlenmeyer flasks and later scaled up to 5 L reactors containing BG11_0_ culture media [46]. They were exposed to 16:8 h of light/dark under a light intensity of 60 μmol photons m^−2^ s^−1^ at 25 °C. Raw cultures were obtained at two different growth phases: logarithmic and stationary. To detect both phases, cyanobacterial concentrations were monitored twice a week by weighing the dry biomass for 22 days. A 40 mL biomass of each strain was filtered using dried (90 °C, 24 h) cellulosic filters. The stationary phase was selected when the concentration of the cyanobacterial cultures remained stable. The stationary phase concentrations were 3 ± 0.08 g L^−1^, 2 ± 0.09 g L^−1^, and 1.6 ± 0.07 g L^−1^ for *N. commune* (day 29), *S. hyalinum* (day 27), and *T. distorta*, (day 27), respectively. The logarithmic phase was chosen when the culture concentration increased at an exponential rate. The logarithmic phase concentrations for *N. commune*, *S. hyalinum*, and *T. distorta* were 1 ± 0.04 g L^−1^ (day 4), 0.8 ± 0.02 g L^−1^ (day 5), and 0.8 ± 0.02 g L^−1^ (day 5), respectively. 

Eighteen experiments on each fungus and oomycete were carried out considering two factors: (i) cyanobacteria treatment (culture, filtrate, and extract) and (ii) growth phase of cyanobacteria (logarithmic and stationary phases); each experiment was conducted in triplicate. The cyanobacteria post-harvest treatments applied were as follows: (i) raw cultures of cyanobacteria (consisting of cyanobacteria cells plus extracellular medium), which were transferred directly from 5 L culturing recipients into 100 mL sterile flasks (culture); and (ii) cyanobacterial filtrates (consisting of extracellular medium without cyanobacteria cells) obtained by filtering cultured cyanobacteria using 7–9 µm pore size and 110 mm diameter cellulosic filters. Cyanobacteria cells were retained in the filters, while filtrates were poured into 100 mL sterile flasks (filtrate), and (iii) cyanobacterial extracts containing extracellular and intracellular media (extract) were obtained following a slightly modified secondary metabolites extraction method [47]: chemical extractants were not used, avoiding the derivative damage of using those. A 50 mL sample of each culture was subjected to sonic disruption for 4 min to break cell walls. Subsequently, they were centrifuged at 5800 rpm for 15 min at 4 °C. Then, the supernatant was filtered in the same way as in the filtrate obtention procedure. All treatments were acquired from working under laboratory axenic conditions.

### 2.4. In Vitro Growth Inhibition Tests

The in vitro growth response of *Phytophtora capsici*, *Pythium aphanidermatum*, and *Fusarium oxysporum* to each of the treatments was assessed by placing 5 mm diameter agar plugs from the edge of actively expanding colonies of the fungus or oomycete downwards in the center of 85 × 15 cm plates in 15 mL of water agar media (WA) (15 g L^−1^ agar, Oxoid Ltd., Basingstoke, UK) amended with the cyanobacterial treatments to be tested. Each cyanobacterial treatment (Table 2) was amended 24 h before fungal placing by pouring 1 mL on the plate just before adding 15 mL of WA cooled to 40 °C. Plates were kept at 4 °C for 24 h. Five plates were inoculated for each fungal isolate and cyanobacteria-based product per trial. Five control plates (only WA) were inoculated at the same time. Inoculated plates were incubated in the dark at 25 ± 1 °C, following a randomized block design, for 3 days for *Pythium aphanidermatum*, 9 days for *Fusarium oxysporum*, and 12 days for *Phytophtora capsici*. Colony diameters were measured at the incubation times mentioned above for each of the pathogens, and the percentage growth (PG) of an isolate on an amended medium was calculated by subtracting the inoculation plug diameter (5 mm) from the diameter of each colony and dividing the average diameter of the amended plates by the average diameter of the unamended control. Percentage growth inhibition (GI) was calculated as GI = 100 − PG. All plates were checked for spores (i.e., oospores and sporangia (*Pythium aphanidermatum*), sporangia (*Phytophtora capsici*), and microconidia (*Fusarium oxysporum*)) with an optical microscope one week after recording colony growth. Each trial was conducted twice, and the data were pooled for statistical analyses.

Initially, raw cultures of the cyanobacteria strains were tested separately in a preliminary trial to check their inhibitory capacity on the three phytopathogenic fungi. For the preliminary trial, only cyanobacterial raw cultures from *N. commune*, *S. hyalinum*, and *T. distorta* obtained at logarithmic phase and concentrated at 4.5 g L^−1^ were used. 

### 2.5. In Planta Bioassays

As our in vitro experiment revealed that *N. commune* exhibited the highest inhibition rates for the three pathogenic fungi, and especially for *P. capsici*, it was selected for bioassay trials to assess the ability of this cyanobacteria to control disease symptoms caused by *P. capsici* on cucumber plants.

Four types of products were tested from raw culture and extracts of the *N. commune* strain obtained at logarithmic and stationary growth phases. Bioassays were arranged on cucumber plants (cv. Super Marketer, Mascarell Semillas, Valencia, Spain) grown on vermiculite (twice autoclaved, 1 h at 120 °C each) at field capacity with a standard nutrient solution (2.1 dS m^−1^). Before sowing, the cucumber seeds were surface disinfected by immersion with 3.5% sodium hypochlorite for 20 min and were subsequently rinsed with tap water and primed individually with the different cyanobacteria-based product by adding a drop until the whole surface of the seed was covered. The seeds were kept in an axenic environment until the drops dried up. Seeds used as control and as reference treatments were not primed. Immediately, seeds were incubated in sterile wet paper at 28 °C in the dark. Only germinated seeds were potted individually in 500 mL containers. The experiment was performed in a growth chamber (14 h photoperiod, >12,000 lux, 23–33 °C). Fertigation with the above-mentioned nutrient solution was applied until the end of the tests according to plant needs, trying to maintain the substrate close to saturation.

In addition to priming, *N. commune* was inoculated by drenching the potting substrate 48 h before sowing cucumbers and repeated 14 days after the first *P. capsici* inoculation. Inoculum consisted of 33 and 66 mL of *N. commune* products (raw cultures and extracts) obtained at the stationary and logarithmic phases, respectively. 

*Phytophthora capsici* strain Mi0211 was inoculated by drenching the potting substrate with 50 mL of inoculum. The inoculum of the pathogen was prepared by grinding several colonies, fully covering the dish surface of the isolate previously grown in PDA in sterile water. The rate was one plate per 300 mL of final suspension. The inoculum density was ca. 2 × 10^4^ CFU per pot. The inoculum consisted mainly of mycelia, and rates were calculated a posteriori employing a dilution plate technique [48] on selective medium P_5_ARP [49]. Pathogen inoculation took place at the cucumber growth stage of two to four true leaves by drenching with 50 mL of inoculum suspension per plant. Inoculation was repeated 23 days later. The reference treatment consisted of plants inoculated with the pathogen without treatments with *N. commune* products. The control consisted of non-inoculated plants watered with an aqueous homogenate of non-colonized PDA. Also, *N. commune* products were evaluated without the addition of pathogen to test their impact on the plants.

For each treatment, one pot with one plant was the elementary replication. Six replications were randomly distributed. Each bioassay included a total of 60 pots with 60 cucumber plants. The bioassays lasted 43 days after the second inoculation with the pathogen (dpi). The temperature and relative humidity in the chamber were measured using an HOBO data logger (Onset Computer Co., Bourne, MA, USA). 

Disease incidence was determined as the percentage of symptomatic plants showing wilting, chlorosis, crown rot, and/or death, and was recorded every three to four days until 43 dpi. This was used to calculate the area under the disease progress stairs (AUDPS) [50]. At the end of the experiments, plants were removed from the pots and the roots examined for symptoms. Then, a disease severity index (DSI) from “0” to “3” was used: 0 = no symptoms; 1 = crown rot; 2 = wilting; 3 = death. Also, the root samples from two randomly assigned pots per treatment were analyzed for *P. capsici* by means of the carnation petals baiting technique [51] to re-isolate the fungus. Negative re-isolation samples were repeated using the four remaining pots. The entire experiment was performed twice over time.

### 2.6. Statistical Analysis

Firstly, one-way ANOVA analysis was applied to find the best mycelial growth inhibitor for each phytopathogen at each studied growth phase. Furthermore, the effect of the growth phase, cyanobacterium strain, and post-harvest treatment on the phytopathogens’ growth inhibition rates were analyzed. All the variables were checked for normality and homogeneity of variance using the Shapiro–Wilk and Levene’s tests, respectively. Data were transformed when it was necessary. Afterward, Tukey’s post hoc test was applied when differences were previously found. Analyses were conducted using R 3.4.2 [52].

For in planta assays, after finding that both trials could be considered statistically equal, the results were analyzed as one individual experiment for more consistent analysis. In addition, since non-inoculated plants showed no disease symptoms, and also plants in treatments with inoculum of the cyanobacterium alone did not differ from those that did not receive any inoculum, statistical analyses were performed with data from treatments that included the pathogen; thus, the disease symptoms were detected. Analysis of variance (ANOVA) was applied to compare treatments for AUDPS and DSI using the statistical software package STATGRAPHIC CENTURION XVI.I (Manugistic Inc., Rockville, MD, USA) for Microsoft Windows (Microsoft, Redmond, DC, USA).

## 3. Results

### 3.1. In Vitro Growth Inhibition Tests

In the preliminary trial for in vitro testing, mycelial inhibition was observed for the three cyanobacteria strains (culture, logarithmic phase). The highest inhibitory effect (71%) was found for *Phytophtora capsici* exposed to *N. commune*, followed by a 41% inhibition rate for *Fusarium oxysporum* against the same strain. *Pythium aphanidermatum* was the most tolerant fungus in this assay, but its growth was also inhibited by *N. commune*. Similarly, *S. hyalinum* acted as a growth inhibitor for *Phytophtora capsici*, an effect that was also observed in the same oomycete when the *T. distorta* culture was applied (Table 3).

**Figure 1:**
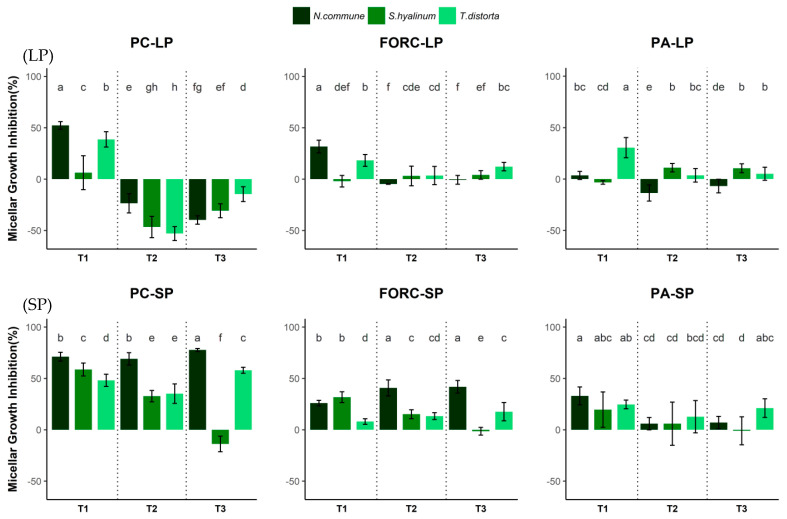
Fungal response to cyanobacteria treatments on logarithmic phase (LP) and stationary phase (SP). Abscissas axis shows treatments used; the ordinate axis shows fungus mycelial growth inhibition induced by cyanobacterial treatments in percentages. Error bars represent standard deviation (*n* = 10, pool of 2 trials with 5 replicates). The letters in the graph correspond to the significant differences between treatments (for each fungus, at one phase) at *p* < 0.05. PC: *P. capsici*; FORC: *Fusarium oxysporum*: *F. oxysporum* f. sp. *radicis-cucumerinum*; PA: *P. aphanidermatum*; T1: raw cultures of cyanobacteria; T2: cyanobacterial filtrates; T3: cyanobacterial extracts.

The complete in vitro trial including all the variations of cyanobacteria confirmed that *N. commune* was the strain with the highest inhibition rates for the three phytopathogens assessed (Figure 1). In any case, any cyanobacteria-based product did not affect fungal or oomycete sporulation; thus, the products tested affected the vegetative skills of the fungi but not their reproductive abilities. The three variables assessed, growth phase, cyanobacterium strain, and post-harvest treatment, involved significant differences for *Phytophtora capsici* and *Fusarium oxysporum*; meanwhile, for *Pythium aphanidermatum*, only cyanobacterium strain, post-harvest treatment, and the combination of strain and growth phase, as well as growth phase and post-harvest treatment, offered significant differences between means (Table 4).

Consistent with the results obtained in the preliminary trial, *Phytophtora capsici* was the most susceptible pathogen to cyanobacteria treatments, and there was mycelial growth inhibition with almost all the cyanobacteria-based products at the stationary phase (excluding *S. hyalinum* extract). At the stationary phase, the greatest mycelial growth inhibition was found for *N. commune* extract (77.70% ± 1.49%) (*p*-value < 0.05) (Figure 1: PC-SP). *N. commune* culture and *N. commune* filtrate (71.13% ± 4.30% and 69.09% ± 5.99%, respectively) were also good inhibitors for *Phytophtora capsici*. *T. distorta* extract also had an important inhibitory effect for this oomycete, with inhibitory percentages higher than *T. distorta* raw culture and filtrate. Although *S. hyalinum* culture and filtrate inhibited the growth of *Phytophtora capsici*, the extract actually promoted mycelial growth. At the logarithmic phase, treatments did not seem to have an inhibitory effect on *P. capsici*, apart from raw cultures of the three strains (Figure 1: PC-LP). 

*Pythium aphanidermatum* seemed to be the most tolerant phytopathogen. Although mycelial growth inhibition was lower than for the other phytopathogens, raw cultures of the three cyanobacteria strains at SP and *T. distorta* raw culture at LP inhibited *P. aphanidermatum* mycelial growth from 20% to 30%, making *N. commune* raw culture the best treatment to control this oomycete. Filtrates and extracts of cyanobacteria showed a tendency toward positive inhibition growth except for *N. commune* filtrate and extract at LP (Figure 1: PA-LP, PA-SP).

The growth phase for the obtention of the cyanobacterium product showed as a key variable in terms of fungal and oomycete mycelial growth inhibition. When comparing the exposition of the three fungi to the three cyanobacteria strains under the different post-harvest treatments (culture, filtrate, and extract), stationary growth phase products showed the best results compared to the logarithmic phase products for *Phytophtora capsici* (*p*-value < 2 × 10^−16^) and *Fusarium oxysporum* (*p*-value < 2 × 10^−16^), but not for *Pythium aphanidermatum* (*p*-value = 0.807). In total, 11 from 19 successful results obtained in the in vitro trials delivered significant differences between growth phases. Those differences were more evident for *Phytophtora capsici* (*p*-value < 2 × 10^−16^) than for *Fusarium oxysporum* (*p*-value = 3.73 × 10^−13^) or *Pythium aphanidermatum* (*p*-value = 1.39 × 10^−05^).

The highest mycelial growth inhibition was found in the stationary phase. Only 5 out of 27 treatments inhibited the phytopathogens’ growth with rates lower than 10%, and *S. distorta* extracts promoted growth of three phytopathogens. In the logarithmic phase, inhibition rates were much lower, with the differences being especially noticeable for the phytopathogen *Phytophtora capsici* (Figure 1: PC-LP vs. PC-SP).

In addition to cyanobacterial strain and growth phase, the influence of the post-harvest treatment applied (culture, filtrate, and extract) was addressed. The multivariate ANOVA test (Table 4) showed that post-harvest treatment itself induced significant differences, and the effect of application of different treatments in inhibition rate was highly influenced by the interaction of growth phase and treatment, and growth phase and cyanobacterium strain for the three phytopathogens, meaning that growth phase and cyanobacterial strain are two variables influencing the effect when different treatments were studied. 

In general, for products obtained at the logarithmic phase, the best inhibitor was the raw culture product, whereas for the stationary phase there was no clear pattern.

### 3.2. In Planta Bioassays

Table 5 shows the results relative to the trials performed for the assessment of the ability of the cyanobacteria *N. commune* strain to control disease symptoms caused by *P. capsici* on cucumber plants. None of the four cyanobacterium products resulted in a significant reduction in disease incidence in relation to reference control infected with the pathogenic oomycete. On the other hand, it should be noted that none of the plants that received treatments in the absence of the pathogen showed disease-related symptoms but very slight brown discoloration of the basal stem was detected in contrast to the control treatment consisting of non-inoculated plants. 

## 4. Discussion

### 4.1. In Vitro Growth Inhibition Tests

The three tested biocrust cyanobacteria strains inhibited the mycelial growth of the phytopathogens, showing different levels of effectiveness. Among them, the *N. commune* strain demonstrated the highest inhibitory potential. Our results agree with previous studies that pointed out that inhibition of mycelial growth strongly depends on the cyanobacteria strain. The studied biocrust cyanobacterial strains showed inhibition rates from 2% to 78%, which fall within the range described by previous studies involving both aquatic and soil cyanobacteria; the reported inhibition rates of mycelial growth were between 10% and more than 80% when applied to control the same phytopathogens [15,21,24,25].

Cyanobacteria culture growth phase at the harvesting step is also a key variable to consider, as higher rates of inhibition were found at the stationary phase (Figure 1). During this growth phase, cyanobacteria go through a range of morphological, metabolic, or transcriptional changes that promote the accumulation of bioactive compounds. Prasanna et al. [53], assessed the potential of cyanobacterial filtrates produced from the biomass harvested at 4 weeks and 8 weeks. They found that phytopathogen inhibition caused by 8 week filtrates was higher than that produced by 4 week filtrates. The authors pointed out an increase in proteins and indolacetic acid (IAA) in the 8 week filtrates compared to the 4 week filtrates, as well as an increase in the hydrolytic activities of different enzymes such as chitosanase and CMCase. Hydrolytic enzymes play an important role in phytopathogen biocontrol: (i) chitosanase hydrolyzes the β-1,4-linkages of chitosan, which is found in the cell walls of fungal phytopathogens [54]; (ii) CMCase is an endoglucanase that hydrolyzes cellulose, which is present in oomycetes [55]. Thus, it may be supposed that in the stationary phase, *N. commune*, *S. hyalinum*, and *T. distorta* are able to release higher amounts of hydrolytic enzymes, causing the degradation of the main compounds of the cell walls in the phytopathogens studied.

Overall, *N. commune* and *T. distorta* were the most effective cyanobacteria against *Phytophtora capsici*, with mycelial growth inhibition that was quite similar using raw culture products at both growth phases. The cell walls of the three phytopathogens assessed in the present study differ in structure and composition. *Pythium aphanidermatum* possesses a cell wall with 18% of cellulose and 82% of (1.3; 1.6) β-glucan; *Fusarium oxysporum* cell walls do not contain cellulose or chitosan [56]; and *Phytophtora capsici* cell walls contain high amounts of cellulose that range from 32% to 35% [57]. The hypothesis that arises from the in vitro assay results and the information found in the literature about the cell wall composition of the three soilborne phytopathogens is the following: cyanobacterial post-harvest treatments contain hydrolytic enzymes such as CMCase that hydrolyze cellulose. As cellulose is most abundant in *P. capsici*, the treatments containing the enzyme CMCase were able to degrade the cell walls of this phytopathogen, while the cell walls of *F. oxysporum* and *P. aphanidermatum* were not affected due to the absence or low quantities of cellulose compared to *P. capsici*.

Hydrolytic enzymes are not the only molecules with phytopathogenic inhibition potential released by the assessed cyanobacteria. Secondary metabolites have been shown to control phytopathogenic fungi. *Nostoc muscorum* filtrate, rich in extracellular metabolites including beta-ionone, norharmone, and α-iso-methyl ionone, reduced the growth of the phytopathogen *Alternaria porri* [41]. Scytonemin A is a metabolite found in *Scytonema*, and it inhibits the growth of various fungi such as *F. oxysporum* [58]. The role of secondary metabolites can be linked with the growth-promoting effect of filtrates and extracts collected at the logarithmic phase, found for all three strains, against *P. capsici*.

Interestingly, the most effective cyanobacteria strains in our research (i.e., *N. commune* and *T. distorta*) were those isolated from the place with the highest degree of erosion: “Gádor quarry”, where nitrogen and carbon contents are lower than in “El Cautivo”. Low nitrogen and carbon content can induce a stress condition in which the cyanobacteria are stimulated to produce a series of secondary metabolites. Strains of *Nostoc* and *Anabaena* genera showed that nitrogen content manipulation leads to increases in certain phenolic compounds. For instance, quinic acid produced by Nostoc 2S7B was increased significantly in nitrogen starvation conditions [59]. 

The effectiveness of soil *N. commune* strains against *Phytophtora capsici* and *Fusarium oxysporum* was reported in another study [20]. In that study, three extractants were used to obtain antifungal products: petroleum ether, methanol, and water. Water extracts had no effect on inhibiting fungus growth, whereas in our research, there was a fungistatic effect when using water extracts. This is a promising result, as raw culture treatments consisting mainly of water and cyanobacteria populations offer a series of advantages: (i) they contribute to avoiding the use of harmful chemicals to obtain the fungistatic products; (ii) they are obtained by employing a time-efficient methodology as raw cultures are applied directly; and (iii) soil inoculation with raw cultures implies a constant source of fungistatic products, as these soil cyanobacteria are well-adapted and are able to survive and continuously produce products that inhibit fungal growth. Indeed, one interesting result for logarithmic phase products when applied to inhibit *Phytophtora capsici* was that only raw culture products acted as biocontrol agents, while with extracts and filtrates colony growth was promoted. In summary, raw culture treatments caused mycelial inhibition, regardless of the growth phase, in almost all experiments. 

*N. commune* stationary phase products could be considered as efficient and environmentally friendly fungistatic products against *Phytophtora capsici* and *Fusarium oxysporum*. These natural biocontrollers are similarly effective in controlling *Phytophtora capsici* as fungicides, with growth inhibition rates of around 77%, but not controlling sporulation like the chemical fungicides pyrimorph or propamocarb [60]; furthermore, they are more effective against *Phytophtora capsici* than other filtrates, as those from some actinobacteria, with an inhibition ratio of 50% [61]. Furthermore, *N. commune* can be cultured in different growth mediums, even in those that are fertilizer-based [62]. 

The results of the in vitro assays allow for the establishment of future lines of research assessing the composition of the stationary post-harvest treatments from *N. commune*, a novel cyanobacteria isolated from soil biocrusts, emphasizing the study of hydrolytic enzymes that act by degrading the cell walls of phytopathogens (e.g., CMCase) or in secondary metabolites with inhibitory effects against phytopathogens.

### 4.2. In Planta Bioassays

The promising results obtained from the in vitro trials for *N. commune* against *P. capsici* were not reproduced in the bioassays with cucumbers. Hence, our initial hypothesis can be assumed for the in vitro experiments; however, when scaled up to in planta bioassays, the hypothesis had to be refuted. This approach highlights the complexity inherent in the use of microorganisms in agriculture for the control of soilborne diseases. The management of soilborne phytopathogens is quite complicated, as these organisms live in the rhizosphere and are able to survive long periods of time to form resistant structures [63].

Our results suggest the need to perform evaluations in the presence of the plant that are beyond in vitro evaluations in which the pathogenic and the beneficial organisms are confronted in the absence of the plant. In this regard, a recent research study [42] evaluated the sonicated extracts obtained from 31 strains of cyanobacteria belonging to 12 different genera (i.e., *Anabaena, Calothrix*, *Dolichospermum*, *Gloeocapsa*, *Leptolyngbya*, *Lyngbya*, *Nodularia*, *Nostoc, Phormidium*, *Synechococcus, Tolypothrix*, and *Trichormus*) for the control of the phytopathogenic oomycete *Pythiun ultimum*, a causal agent of damping-off in cucumber seedbeds. The study showed that approximately one-third of all the cyanobacterial extracts showed some ability to delay the growth of *P. ultimum*. Of these, when in planta evaluations were performed, only one was positioned as an effective control agent against damping-off caused by *P. ultimum* in cucumber seeds after biopriming. 

It should be noted that, in many cases, even when satisfactory results are obtained in controlled environment chamber evaluations, experiments should be addressed under real growing conditions (e.g., greenhouse soils) in order to determine a practical efficacy for use in agriculture. In this case, the complexity would be even greater and the variability of the results would probably depend on a multitude of casuistries not covered by in vitro studies nor those carried out under controlled conditions [64].

There are multiple methodological approaches that influence, in great measure, the success of the results when scaling up the in vitro tests. When scaling up from in vitro to in planta assays, positive inhibitory results have been found in the literature, and each one follows a different methodological approach (among them and compared with the present study). Some factors that can influence the success of the scaling up are the following: (i) seed sowing time in contact with the treatment [38]; (ii) ratio of inoculum to phytopathogen [39]; (iii) area of cyanobacterial treatment application, where some biocontrol experiments focused on the treatment or fungi application in the leaves [40,41] and others (present study, [38,39]) in the soil; or (iv) applying treatment after or before the phytopathogen inoculation [41]. Also, the cyanobacterial inoculum dose and the application strategy in greenhouse trials need to be deeply evaluated to ensure the appropriate contact between biocontrol agent and pathogen for achieving the efficiency and success of the inoculant [65].

Using native cyanobacteria isolated from biocrusts as biocontrol agents is an advantage, as they that can be directly inoculated in the soil, generating a micro-ecosystem that provides nutrients to the plants (C and N), and other compounds such as phytohormones [66] are a novelty worthy of future research in scaling up experiments. In this regard, future lines of research should focus on the application methods of the products at in planta trial level, as the *N. commune* post-harvest treatments in the stationary phase have been proven to act as biocontrol agents, especially for *P. capsici*. 

## 5. Conclusions

*N. commune*, *S. hyalinum*, and *T. distorta*, biocrust cyanobacteria native to soils, have shown moderate to high natural antagonism for three phytopathogenic fungi and oomycetes for different cyanobacteria products. The highest antagonism was observed for raw cultures, filtrates, and extract treatments of these cyanobacteria collected at the stationary phase, in contrast to cultures obtained at the logarithmic phase. The most promising phytopathogen–cyanobacterium combination was *P. capsici* × *N. commune* strain CANT2 UAM817. Raw culture of this strain was the most fungistatic among all tested treatments against *P. capsici* and *Fusarium oxysporum* f. sp. *radicis-cucumerinum*, regardless of the growth phase, and could constitute an inexhaustible source of fungistatic products in soils as this strain is able to survive and grow in soils where they are well adapted [43].

The role of cyanobacteria in soils seems to be more important for other microorganisms in close proximity, yet their contribution to control plant pathogens remains unclear. Factors such as species selection, compound screening, detailed methods for their in planta application, and assessment require further detailed investigation. This study also highlights the importance of scaling up experiments from in vitro to in planta assays to account for the complexity of real field settings.

## Figures and Tables

**Table 1:** In vivo assays of cyanobacterium strains tested to control plant pathogens.

Cyanobacterium	Phytopathogen	Plant	References
*Anabaena laxa* RPAN8	*Fusarium solani* ITCC 6731 *Rhizoctonia solani* ITCC6180	Tomato	[26]
*Anabaena minutissima* BEA0300B	*Botrytis cinerea* 06 *Rhizoctonia solani* DAFS3001	Tomato	[37,38]
*RPAN8 A. laxa* RPAN59 *A. variabilis.*	*Fusarium oxysporum* f. sp. *lycopersici* (ITCC 4998) *Fusarium moniliforme* (ITCC 4223)	Tomato	[39]
*Anabaena* sp. BEA0300B.	*Podosphaera xanthii*	Zucchini	[40]
*Anabaena oryzae*, *Arthrospira* sp., *Nostoc minutum*, *Nostoc muscorum* *Oscillatoria* sp.	*Alternaria porri*	Onion	[41]
*Tolypothrix* sp. SAB-M465 *Anabaena* sp. SAB-B912	*Pythium ultimum* CECT 2365	Cucumber	[42]

**Table 2:** Factors assessed in the in vitro trials.

Factor 1: Cyanobacterium Strain	Factor 2: Growth Phase	Factor 3: Product Treatment
*Nostoc commune*	Stationary	Raw ‘living’ cultures
*Scytonema hyalinum*	Logarithmic	Filtrated cultures
*Tolypothrix distorta*		Sonicated cultures

**Table 3:** Results of the preliminary trial: inhibitory effect caused by raw cultures for each cyanobacteria strain on the three phytopathogens under study. Data show the percentage inhibitory growth effect (mean ± standard deviation), considering 10 replicates per *cyanobacteria x phytopathogen* combination (*n* = 10).

	Cyanobacteria Strain		
Target Phytopathogen	*N. commune*	*S. hyalinum*	*T. distorta*
*P. capsici*	71 ± 7	61 ± 7	57 ± 16
*F. oxysporum*	41 ± 9	13 ± 9	11 ± 4
*P. aphanidermatum*	32 ± 9	3 ± 20	7 ± 14

**Table 4:** *p*-values of the three-way ANOVA performed to test the effect of the factors on the dependent variable (mycelial inhibition). Factors are cyanobacterium strain, growth phase, treatment, and the interaction between them; *p*-value > 0.05. * Illustrates significant differences.

Factor	*P. capsici*	*F. oxysporum*	*P. aphanidermatum*
*Cyanobacterium* strain	<2 × 10^−16^ *	<2 × 10^−16^ *	4.38 × 10^−10^ *
Growth phase	<2 × 10^−16^ *	<2 × 10^−16^ *	0.807
Treatment	<2 × 10^−16^ *	2.05 × 10^−6^ *	0.001 *
*Cyanobacterium* strain * Growth phase	<2 × 10^−16^ *	3.73 × 10^−13^ *	1.39 × 10^−05^ *
*Cyanobacterium* strain * Treatment	8.86 × 10^−12^ *	4.18 × 10^−06^ *	0.208
*Growth phase* * Treatment	5.50 × 10^−13^ *	3.60 × 10^−06^ *	0.031 *
Growth phase * *Cyanobacterium* strain * Treatment	<2 × 10^−16^ *	<2 × 10^−16^ *	0.208

**Table 5:** Effects of *P. capsici* pathogenic strain Mi0211 on cucumber cv. Super Marketer under controlled conditions depending on the treatment (raw culture or extract) and growth phase (logarithmic and stationary) of the cyanobacterium *N. commune* strain CANT2 UAM817 previously inoculated, and the reference treatment consisted of pathogen inoculation with no cyanobacterium inoculum. The results correspond to the average of the two trials carried out over time (*n* = 10, pool of 2 trials with 5 replicates).

Treatment	Growth Phase	Pathogen	%Symptomatic Plants	^a^ AUDPS (Symptoms)	% Dead Plants	^b^ AUDPS (Death)	^c^ DSI
raw culture	logarithmic	yes	100.0	29.88	16.7	1.92	1.83
extract	logarithmic	yes	83.3	35.46	25.0	6.21	2.33
raw culture	stationary	yes	91.7	47.54	33.3	7.67	2.67
extract	stationary	yes	100.0	51.83	50.0	13.75	2.42
reference		yes	100.0	45.21	33.3	8.38	2.17
		*p*-value		0.4842		0.2017	0.6483

Absence of letters in the same column indicate no differences among inocula treatments. ^a^ Disease incidence is expressed as area under disease progress stairs (AUDPS) calculated from symptomatic plants. ^b^ Disease incidence is expressed as area under disease progress stairs (AUDPS) calculated from dead plants. ^c^ Disease incidence was measured as root damage at the end of the experiments and expressed using a disease severity index (DSI) ranging from 0 to 3 (0 = no symptoms; 1 = crown rot; 2 = wilting; 3 = death).

## Data Availability

The data presented in this study are available within the article. For more details, readers are invited to directly ask the corresponding authors.
